# Egyptian propolis extract for functionalization of cellulose nanofiber/poly(vinyl alcohol) porous hydrogel along with characterization and biological applications

**DOI:** 10.1038/s41598-023-34901-6

**Published:** 2023-05-12

**Authors:** Safaa Saleh, Ahmed Salama, Amira M. Ali, Ahmed K. Saleh, Bothaina Abd Elhady, Emad Tolba

**Affiliations:** 1 Department of Physics, Faculty of Science, Al-Azhar University, (Girls Branch), P.O Box 11884, Cairo, Egypt; 2 Cellulose and Paper Department, National Research Centre, 33 El-Bohouth St., Dokki, P.O. 12622, Giza, Egypt; 3 Polymers and Pigments Department, National Research Centre, 33 El-Bohouth St., Dokki, P.O. 12622, Giza, Egypt

**Keywords:** Chemical biology, Hydrology, Chemistry, Materials science

## Abstract

Bee propolis is one of the most common natural extracts and has gained significant interest in biomedicine due to its high content of phenolic acids and flavonoids, which are responsible for the antioxidant activity of natural products. The present study report that the propolis extract (PE) was produced by ethanol in the surrounding environment. The obtained PE was added at different concentrations to cellulose nanofiber (CNF)/poly(vinyl alcohol) (PVA), and subjected to freezing thawing and freeze drying methods to develop porous bioactive matrices. Scanning electron microscope (SEM) observations displayed that the prepared samples had an interconnected porous structure with pore sizes in the range of 10–100 μm. The high performance liquid chromatography (HPLC) results of PE showed around 18 polyphenol compounds, with the highest amounts of hesperetin (183.7 µg/mL), chlorogenic acid (96.9 µg/mL) and caffeic acid (90.2 µg/mL). The antibacterial activity results indicated that both PE and PE-functionalized hydrogels exhibited a potential antimicrobial effects against *Escherichia coli*,* Salmonella typhimurium, Streptococcus mutans*, and *Candida albicans*. The in vitro test cell culture experiments indicated that the cells on the PE-functionalized hydrogels had the greatest viability, adhesion, and spreading of cells. Altogether, these data highlight the interesting effect of propolis bio-functionalization to enhance the biological features of CNF/PVA hydrogel as a functional matrix for biomedical applications.

## Introduction

The most pronounced application of three-dimensional (3D) tissue-like biocompatible materials is to direct tissue regeneration or healing after damage. This relies on the ability of these materials to optimize the physiological microenvironment by using biochemical, biophysical cues, and sometimes mechanical stimulation to enhance cell function^1,2^. Indeed, bioactive materials play multiple prominent roles in triggering cell proliferation and differentiation, as well as minimizing inflammation response that can delay the healing process^3,4^. Hydrogels are smart biomaterials that can be applied for the healing of a variety of tissues, such as skin, cartilage, bone, and blood vessels^5^. They can provide an optimal (3D) structure similar to native extracellular matrix (ECM) and allow diffusion of gases, nutrients, and waste through the elastic crosslinked networks^6^. Over the past decades, a variety of polymeric materials of either natural or synthetic origin have been used to develop functional hydrogels. Fiber-reinforced hydrogels are a class of composite hydrogels in which the gel networks are usually reinforced with fiber structures to improve the mechanical performance and also constrain the swelling behavior^7–9^.

Cellulose is the most abundant naturally derived polymer on earth, it’s the main component of plant cell walls and a few animal cells^10^. It is a linear homopolysaccharide consisting of β-d-anhydroglucopyranose units, linked by β (1→4) ether bonds (glycosidic links). The formed cellulose chains are linked by hydrogen bonds to form fibrils that consist of amorphous and crystalline regions. CNF signifies a specific class of nanocelluloses that composed of amorphous and highly ordered domains alternately associated and are typically obtained through the mechanical disintegration of cellulose fibrils^11,12^. As a result, CNF has been emerging nano-size biomaterials that display highly strength, surface area, and tunable surface chemistry, allowing for controlled interactions with polymers, nanoparticles, small molecules, and biological materials. For example, CNF was embedded in an alginate and polyvinyl alcohol solution to form a stable hydrogel that promotes in situ mineralization of calcium phosphates^13^. Also, 2,2,6,6-tetramethylpiperidine-1-oxyl (TEMPO)-oxidized CNF, which has carboxyl groups, was grafted by soy protein hydrolysate via amidation of carboxylic groups. The grafted CNF assisted the hydroxyapatite mineralization from twice-simulated body fluid to form a new bioactive material^14^.

One of the hydrophilic polymers used to produce biocompatible synthetic hydrogels is PVA, a semi-crystalline polymer. PVA hydrogel can be physically cross-linked to produce porous biocompatible materials with microstructure and deformation properties comparable to biological membranes, making them ideal for biomedical applications^15,16^. Physical crosslinking of PVA can be performed by repeated freezing and thawing cycles. In this method, the prepared PVA solution is frozen, which allows ice crystals to form pushing away the PVA chains and packing them together in confined spaces. Crystallization of the polymer happens in these regions. Then, through the thawing process, the ice crystals melt, forming porous cryogels with crystals working as crosslinking junctions^17^. Although traditional hydrogels possess excellent properties such as safety, biocompatibility, and higher hydrophilicity, they have limited biological performance. Today, the combination of hydrogel with therapeutic agents (i.e., drugs, natural extracts, functional proteins, or genes) represents an effective method to offer more favorable environments for the tissue regeneration process and to avoid the rapid metabolism of therapeutic agents^18^.

Propolis is a natural byproduct of beekeeping that has been used for wound healing since ancient times. More than 300 compounds are present in propolis, and the colour varies with the regions vegetation and the plant source. Depending on the regional vegetation, it can be yellowish-green or dark brown^19^. As well as securing cracks in hives, propolis strengthens honeycomb cells and protects hives from microbial invasions^20^. The main polyphenolic components of propolis are flavonoids and phenolic acid esters, followed by aromatic acids, lignans, and terpenoids^21^. Usually, bee wax makes up 30% of the mixture, pollen makes up 5%, and resins and vegetable balsams make up 50%^21^. In addition to balsamic, resinous, and gummous substances, propolis also contains pollen, saliva, and wax, and is derived from the exudates and sprouts of plants by honey bees^22^.

The antimicrobial properties of propolis are attributed to flavonoids, which are effective against Gram-positive and Gram-negative bacteria^23^. Many biological properties are associated with propolis, including antibacterial, antifungal, antiviral, anti-inflammatory, anticancer, and antitumor properties^24,25^. Propolis-based materials have been applied as wound healing materials. For example, propolis as a cream is used for enhancement of dermal tissue healing and reduce the wound inflammation better than silver sulfadiazine^26^. It promotes the proliferation, activation, and growth capacity of skin cells, with no toxicity or allergic reactions.

**Figure 1:**
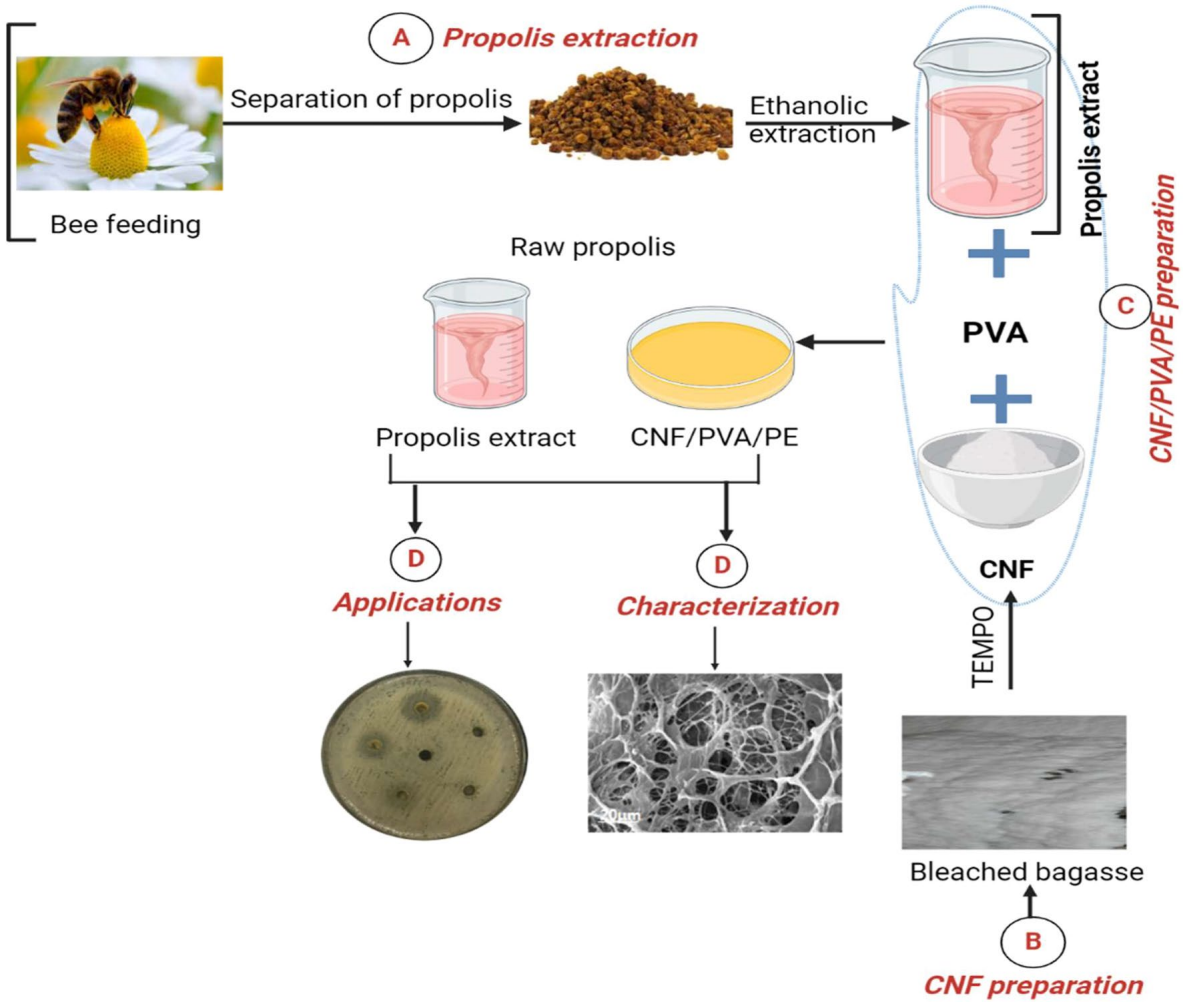
Schematic representation of the five main steps in the current work: (**A**) propolis extract; (**B**) CNF preparation based on bleached bagasse; (**C**) CNF/PVA loaded with PE; (**D**) microstructure characterization of obtained samples; and (**D**) biological applications.

Recently, functional hydrogels have attracted huge attention for tissue engineering and regeneration applications due to their hydrophilicity, flexibility, and inherent adhesive potential to biological tissues. These distinctive characteristics play a critical role in increasing the residence time of a given therapeutic ingredient in direct contact with its biological target. Therefore, the present study aims to prepare a 3D hydrogel from PVA and CNF using the repeated freezing and thawing cycles, as a safe physically cross-linked hydrogel for effective combination of PE without undesired side interactions with cross-linkers that are involved in chemically cross-linked hydrogels. The microstructure features of PE-loaded hydrogels were analyzed through Fourier transform infrared spectroscopy (FT-IR), SEM, and X-ray diffraction (XRD). In addition, in vitro test cell viability and attachment to human normal fibroblast cells (HFB4) were determined. Finally, antibacterial and anti-inflammation potentials were also investigated, as summarized in Fig. 1.

## Results and discussions

### Polyphenol compounds determination of PE

**Figure 2:**
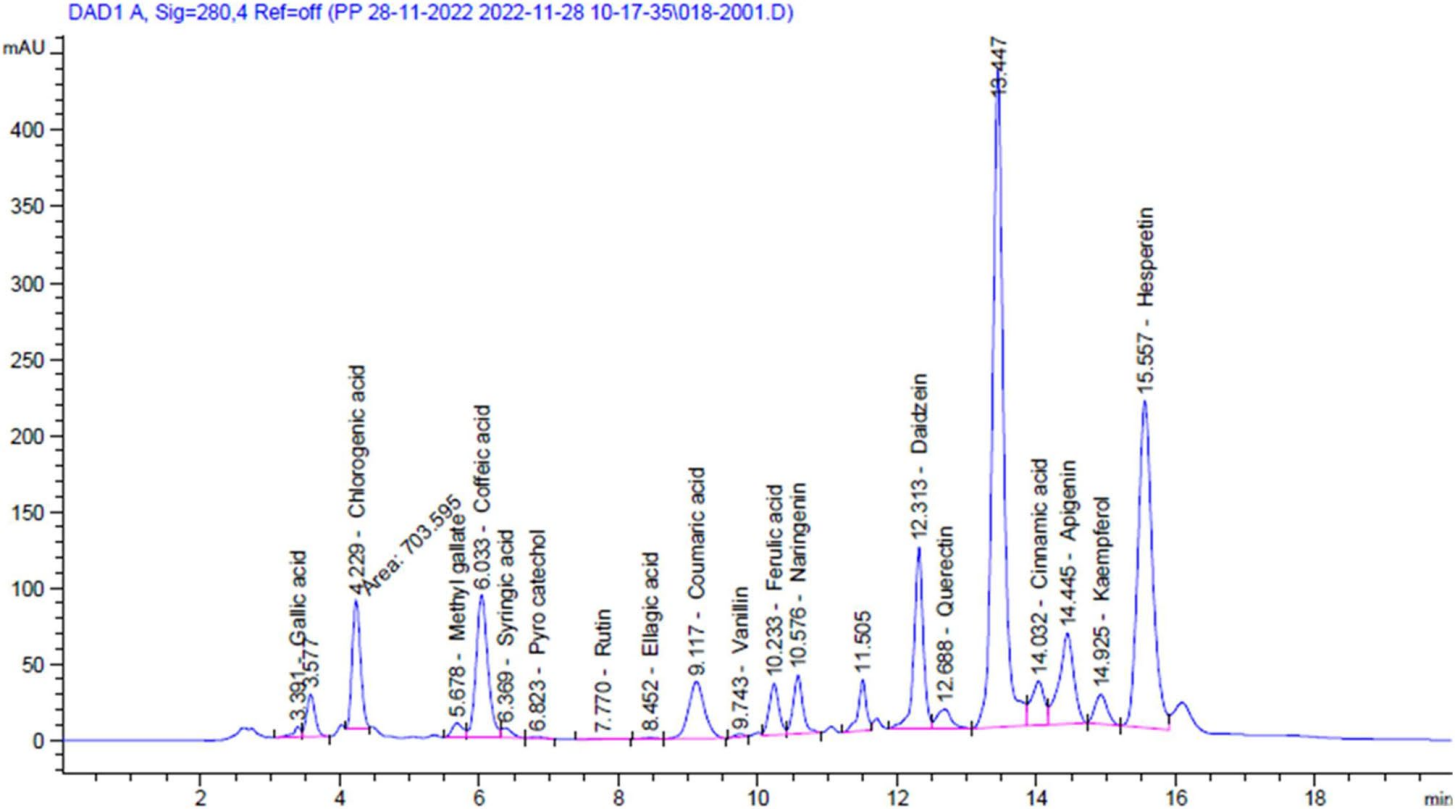
HPLC fingerprint of PE.

The chemical composition of PE may vary depending on the honey bee’s food source. Therefore, it is important to illustrate the chemical composition of PE to emphasise the presence or absence of certain compounds. As shown in Table 1 and Fig. 2, the HPLC detection method was able to identify 18 polyphenol compounds in PE, which play extremely important roles in its biological activity. The polyphenol compounds with highest content illustrated in PE were hesperetin (183.73 µg/mL), chlorogenic acid (96.92 µg/mL), caffeic acid (90.28 µg/mL), daidzein (67.89 µg/mL) and apigenin (66.59 µg/mL). While the rutin and vanillin had low concentrations of 0.8 and 0.7 µg/mL, respectively. On the other hand, the only catechin not observed in the PE when compared with the standard. Geographical origin and extraction solvent of propolis are the main factors that affect the availability and concentrations of polyphenol compounds, as illustrated in Table 2.

**Table 1:** The names, retention time, area, and concentrations of Polyphenol compounds of PE.

No.	Compound	Retention time (min)	Area	Area (%)	Concentration (µg/mL)
1	Gallic acid	3.39	52.02	0.359	4.35
2	Chlorogenic acid	4.22	703.59	4.857	96.92
3	Catechin	4.60	0.00	0.00	0.00
4	Methyl gallate	5.67	105.75	0.730	5.92
5	Caffeic acid	6.03	1097.84	7.578	90.28
6	Syringic acid	6.36	68.44	0.472	4.98
7	Pyro catechol	6.82	12.69	0.087	1.88
8	Rutin	7.77	6.94	0.047	0.82
9	Ellagic acid	8.45	10.50	0.072	2.36
10	Coumaric acid	9.11	596.94	4.120	18.08
11	Vanillin	9.74	16.10	0.111	0.71
12	Ferulic acid	10.23	326.96	2.257	22.07
13	Naringenin	10.57	363.67	2.510	43.64
14	Daidzein	12.31	1087.63	7.508	67.89
15	Quercetin	12.68	181.25	1.251	21.14
16	Cinnamic acid	14.03	384.07	2.651	7.39
17	Apigenin	14.44	864.36	5.967	66.59
18	Kaempferol	14.92	235.33	1.624	18.11
19	Hesperetin	15.55	3098.45	21.38	183.73

**Table 2:** Geographical and extraction solvent of propolis effect on the availability of polyphenol compounds.

Geographical origin	Extraction solvent	Number of polyphenol compound	Main polyphenol compound	Reference
Egypt	Ethanol	18	Hesperetin, chlorogenic acid, caffeic acid, daidzein and apigenin (66.59 µg/mL)	Current study
China	Ethanol/water	20	Pinobanksin 3-O-acetate, pinocembrin, chrysin, galangin, and pinobanksin	^27^
Italy	Hydrochloric	40	caffeic acid, p-coumaric acid, ferulic acid isoferulic acid, and 3,4-dimethyl caffeic acid	^28^
Egypt	Ethanol	12	Gallic acid, rutin, kaempferol, Coumaric acid	^29^
Brazil	Ethanol and Dichloromethane	18	Cinnamic acid, flavonoids, benzoic acid and a few benzoates, non-hydroxylated aromatics, and aliphatic acids	^30^
Turkey	Water	17	Caffeic acid, trans-cinnamic, chlorogenic, and caffeoylquinic acids	^31^
	Ethanol		Chrysin, caffeic acid phenethyl ester, pinocembrin, galangin, naringenin	
Brazil	Ethanol	10	Liquiritigenin, calycosin, formononetin, and biochanin	^32^
Algeria	Ethanol	4	Chlorogenic acid, caffeic acid, gallic acid, and p-coumaric acid	^33^
Argentina	Ethanol/water	7	Cinnamic acid, caffeic acid prenyl ester, caffeic acid 3,4-dihydroxyphenethyl ester, liquiritigenin, 2′,4′-dihydroxychalcone and 2′,4′-dihydroxy-3′-methoxychalcone	^34^

### Characterizations of CNF

**Figure 3:**
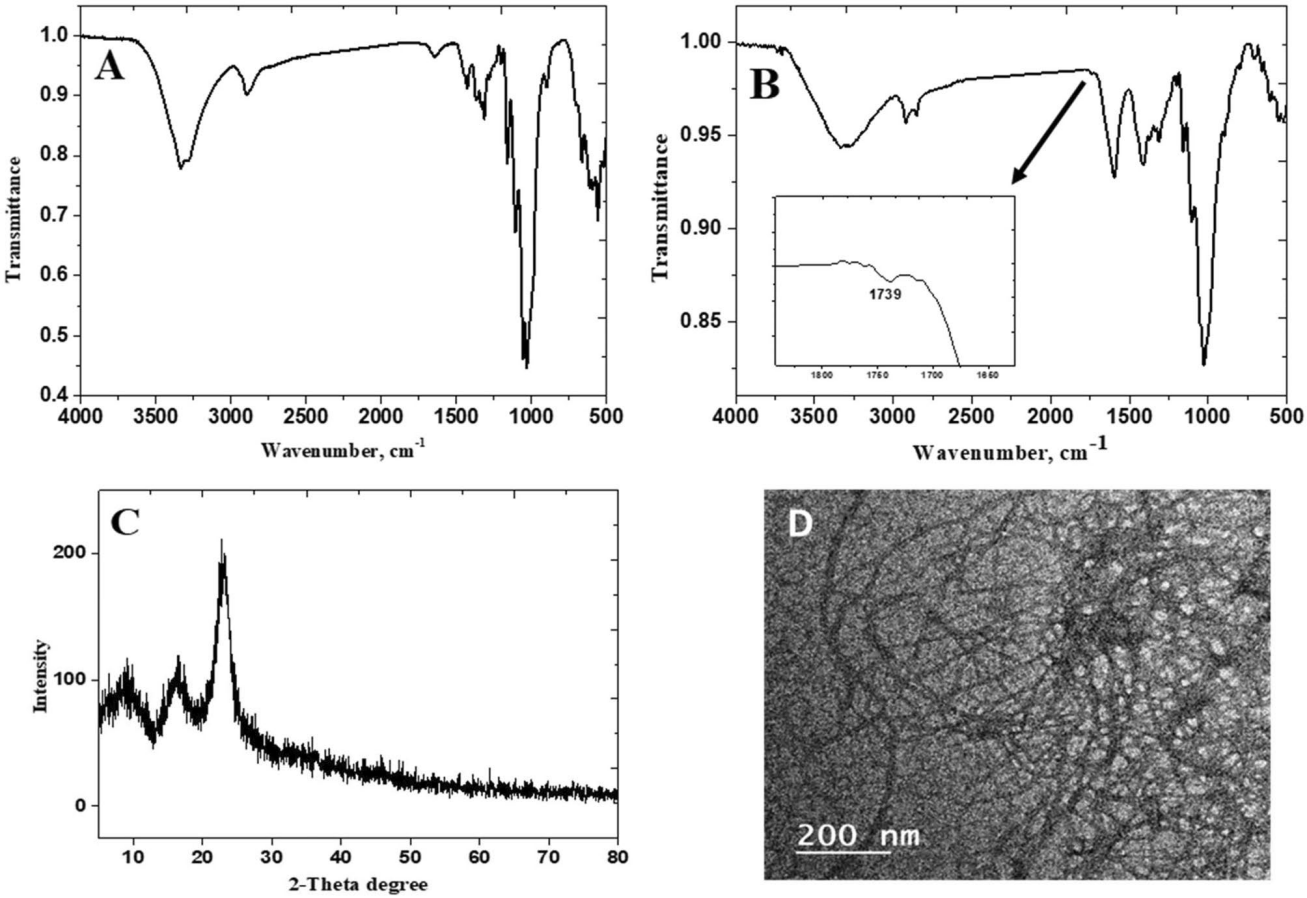
Chemical analysis of cellulosic materials (**A**) FT-IR of cellulose, (**B**) FT-IR of CNF, (**C**) XRD of CNF and (**D**) TEM image of CNF.

For the selective oxidation of primary hydroxyl groups in cellulose (Fig. 3A), TEMPO-mediated oxidation is a suitable method for C-6 oxidation to generate a reactive carboxylic groups. Figure 3B shows the FT-IR spectrum of CNF, which exhibits the characteristic peaks for cellulose chains^35^. A new band, however, was seen at 1739 cm^−1^ that was attributed to the stretching of carbonyl groups (C=O), from the TEMPO oxidation process. The prepared CNF was confirmed by the XRD pattern reported in Fig. 3C. It shows two main diffraction peaks at 16.4° (110) and 23.1° (200) which are characteristic of native cellulose. Also, the crystallinity percentage was estimated according to the Segal method and recorded ∼ 73%^36^. The internal structure of CNF was also examined using transmission electron microscope (TEM) analysis. Figure 3D showed CNF with a micrometric length and a diameter varying from 4 to 20 nm, calculated via image analysis. Moreover, the nanofibers exhibited a uniform structure due to the homogeneous formation of carboxylate groups on the surface of the fibers.

### Microstructure features of the prepared fiber-reinforced hydrogels

**Figure 4:**
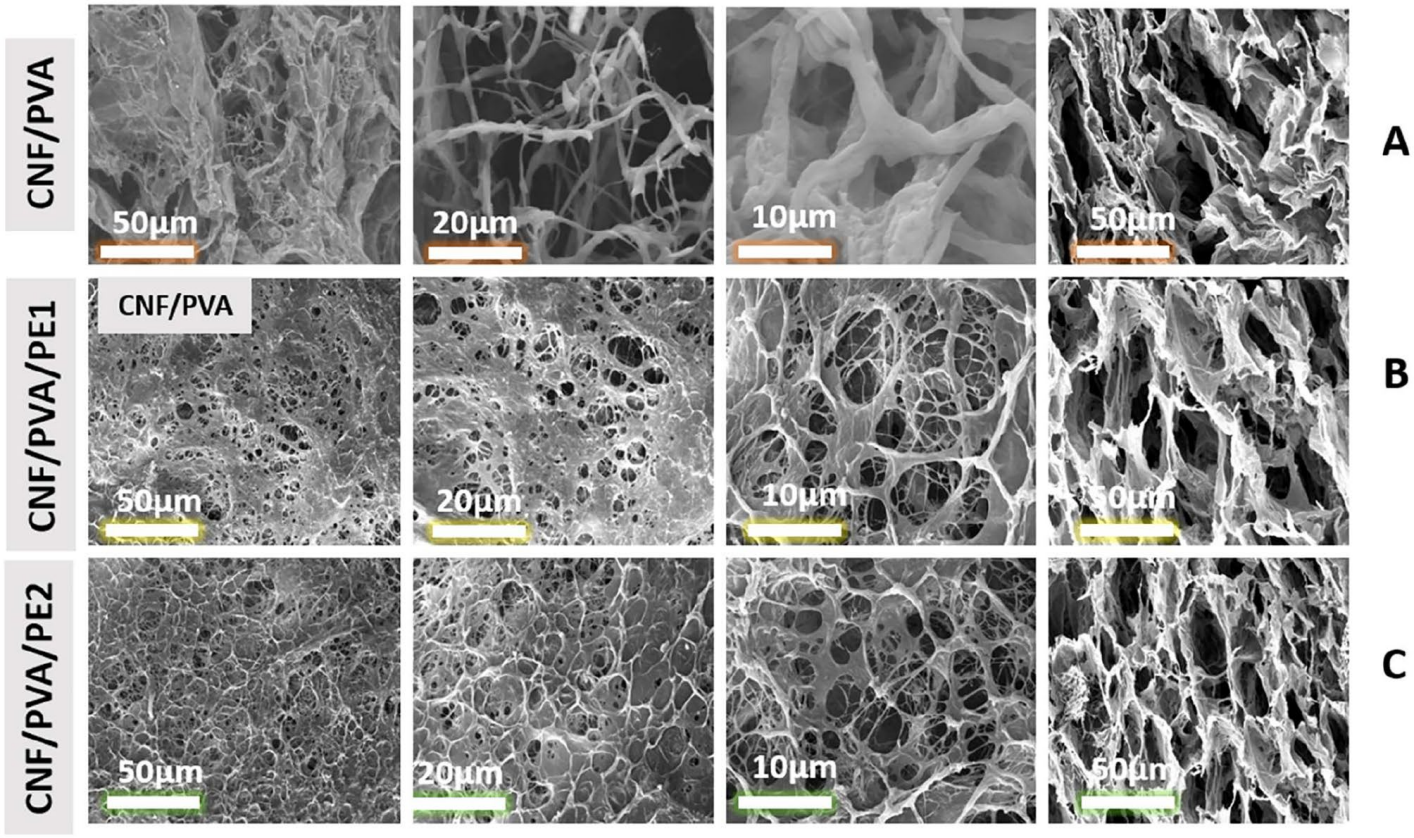
SEM images of fiber reinforced hydrogels at different magnifications (×200, at ×500 and ×1000) and related cross-section images of the same sample (×200). (**A**) CNF/PVA, (**B**) CNF/PVA/PE1, and (**C**) CNF/PVA/PE2.

Indeed, polymer hydrogels are fabricated by physical or chemical cross-linking, or even both. Physically cross-linked hydrogels are obtained through weak interactions, while chemical covalent bonding is formed when hydrogels are cross-linked chemically. Among them, PVA hydrogels can be simply formed by the freeze-thawing technique. In this study, PE-loaded hydrogels based on CNF and PVA were prepared by simple physical crosslinking after successive runs of freezing and thawing followed by freeze drying to produce porous 3D structures. Figure 4A–C illustrates SEM images of the surfaces of the CNF/PVA, CNF/PVA/PE1 and CNF/PVA/PE2 fiber reinforced hydrogels, along with the cross section images for the same hydrogel sample. Figure 4D shows the cross-section images for the same hydrogels. Images of cross section and surface confirm the formation of 3D rough interconnected porous structures with irregular pores varying in size from 10: 100 µm. This microporous structure is very important for cell recruitment because it facilitates the transport of oxygen and nutrients. The introduction of PE into the hydrogel results in a decrease in pore size and surface roughness. This decrease in pore size suggests the presence of PE on the surface of the hydrogel which promotes its fast release when implanted in vivo. This fast release of antibacterial agents such as PE is important to stop the bacterial invasion.

**Figure 5:**
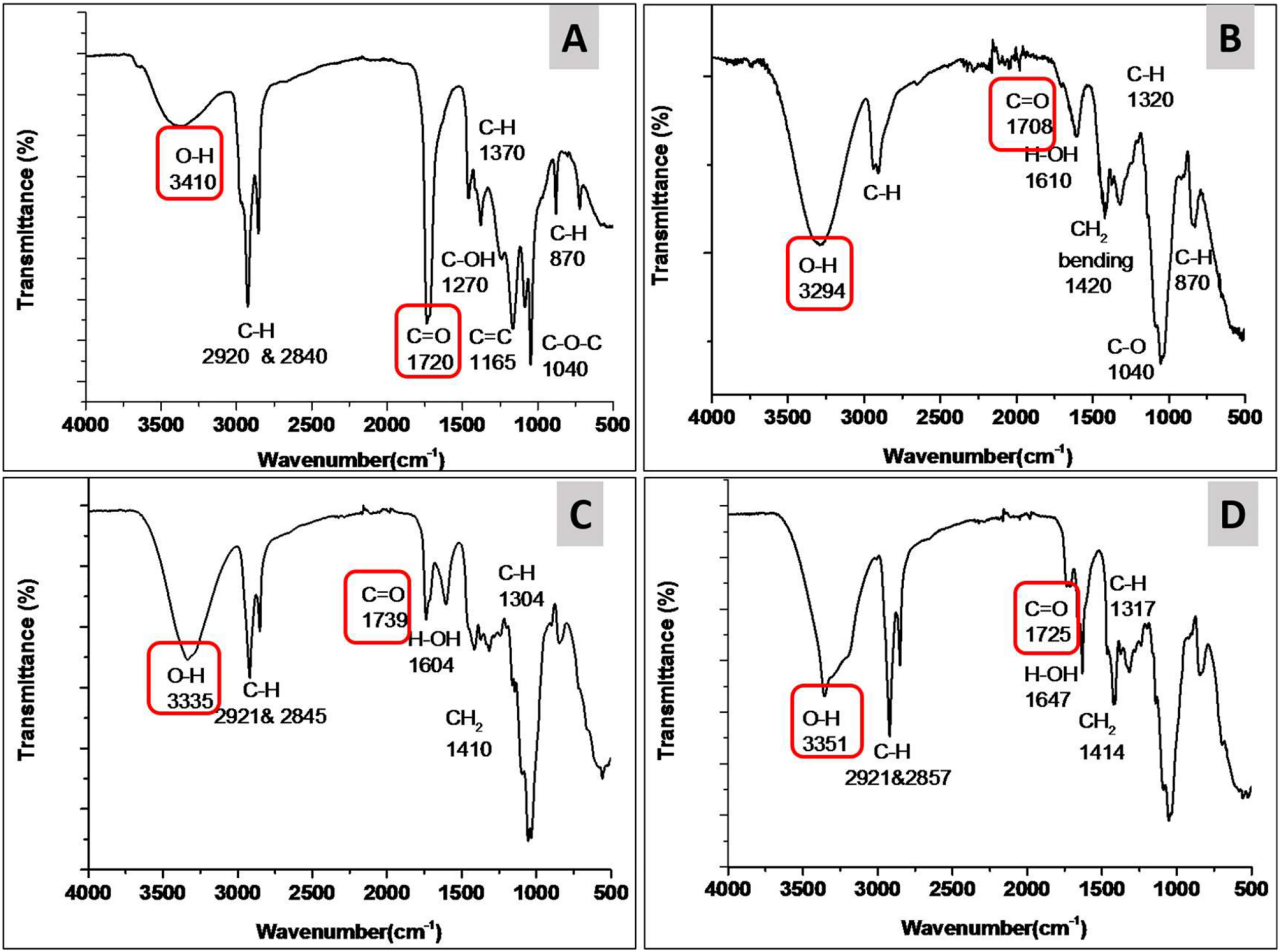
FT-IR spectra of (**A**) PE, (**B**) CNF/PVA, (**C**) CNF/PVA/PE1 and (**D**) CNF/PVA/PE2 fiber reinforced hydrogels.

Figure 5 illustrates the FT-IR spectra of PE and PE-loaded PVA/CNF hydrogel samples. The IR spectrum of the PE sample, Fig. 5A, shows a broad band centered at 3410 cm^−1^, which is typically attributed to the O–H stretching vibration of the phenol compounds^37^. The two bands at 2920 and 2840 cm^−1^ are related to C–H asymmetric and symmetric stretching of hydrocarbon compounds, respectively^38^. While the bands at 1720 and 1460 cm^−1^ are assigned to the carbonyl C=O stretching vibration of flavonoid and lipid content. Furthermore, the bands at 1370 and 1271 cm^−1^ are due to scissor vibrations of C–H groups and C–O–H stretching vibrations, respectively^39^. Finally, the bands at 1165, 1040, and 870 cm^−1^ are related to the stretching vibration of alkenes C=C bond, the aromatic ether C–O–C bond and the C–H wagging vibration of phenolic compounds^40^.

On the other hand, the FT-IR spectrum of CNF/PVA hydrogel shows a broad band at 3294 cm^−1^, which is due to the O–H stretching vibration of hydroxyl group of both PVA and CNF, as shown in Fig. 5B. The bands at 2905, 1708 and 1420 cm^−1^ belong to C–H stretching, C=O stretching, and CH_2_ bending vibrations, respectively. The other bands at 1040 and 870 cm^−1^ are related to the C–O and CH_2_ stretching vibrations^41^. The FT-IR spectra of CNF/PVA/PE1 and CNF/PVA/PE2 fiber reinforced hydrogels are shown in Fig. 5C and D, respectively. The spectra of both samples exhibit peaks related to CNF/PVA hydrogel, with some modifications. For instance, the O–H stretching bands were shifted to 3335 and 3351 cm^−1^ for the CNF/PVA/PE1 and CNF/PVA/PE2 samples, respectively. Moreover, the C=O stretching bands were observed at 1739 cm^−1^ for CNF/PVA/PE1 sample and at 1725 cm^−1^ for the CNF/PVA/PE2 sample. Together, these shifts can be attributed to the interaction between the CNF/PVA polymer matrix and propolis^41^.

### Water uptake

**Figure 6:**
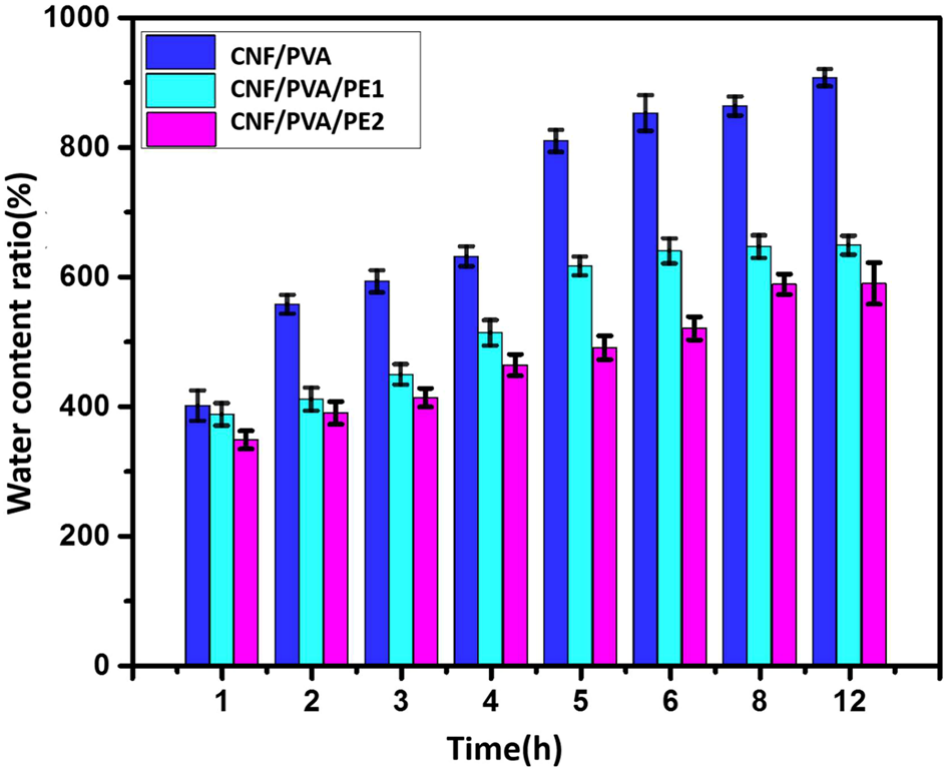
Water content ratio (%) of samples CNF/PVA, CNF/PVA/PE1, and CNF/PVA/PE2 at different time intervals.

The tendency to water uptake or swelling behavior of CNF/PVA, CNF/PVA/PE1, and CNF/PVA/PE2 fiber reinforced hydrogels is illustrated in Fig. 6. The ratios of water uptake for all samples are high and increased gradually with time. This may refer to the high porous structure of the samples, which allows water to diffuse easily through the hydrogel. As shown in Fig. 6, the samples loaded with PE (CNF/PVA/PE1 and CNF/PVA/PE2) exhibit lower water uptake ratios than those of neat (CNF/PVA). For example, at a time interval of 12 h, the ratio of water uptake for CNF/PVA is about 907%, while for CNF/PVA/PE1 and CNF/PVA/PE2 are 648% and 590%, respectively. Therefore, the presence of PE in the CNF/PVA/PE1 and CNF/PVA/PE2 fiber reinforced hydrogels diminishes their ability to water uptake which may be attributed to the hydrophobic nature of PE and the decrease in pore size as indicated from SEM observations (Fig. 4)^40^.

### Assessment of antimicrobial activity

**Figure 7:**
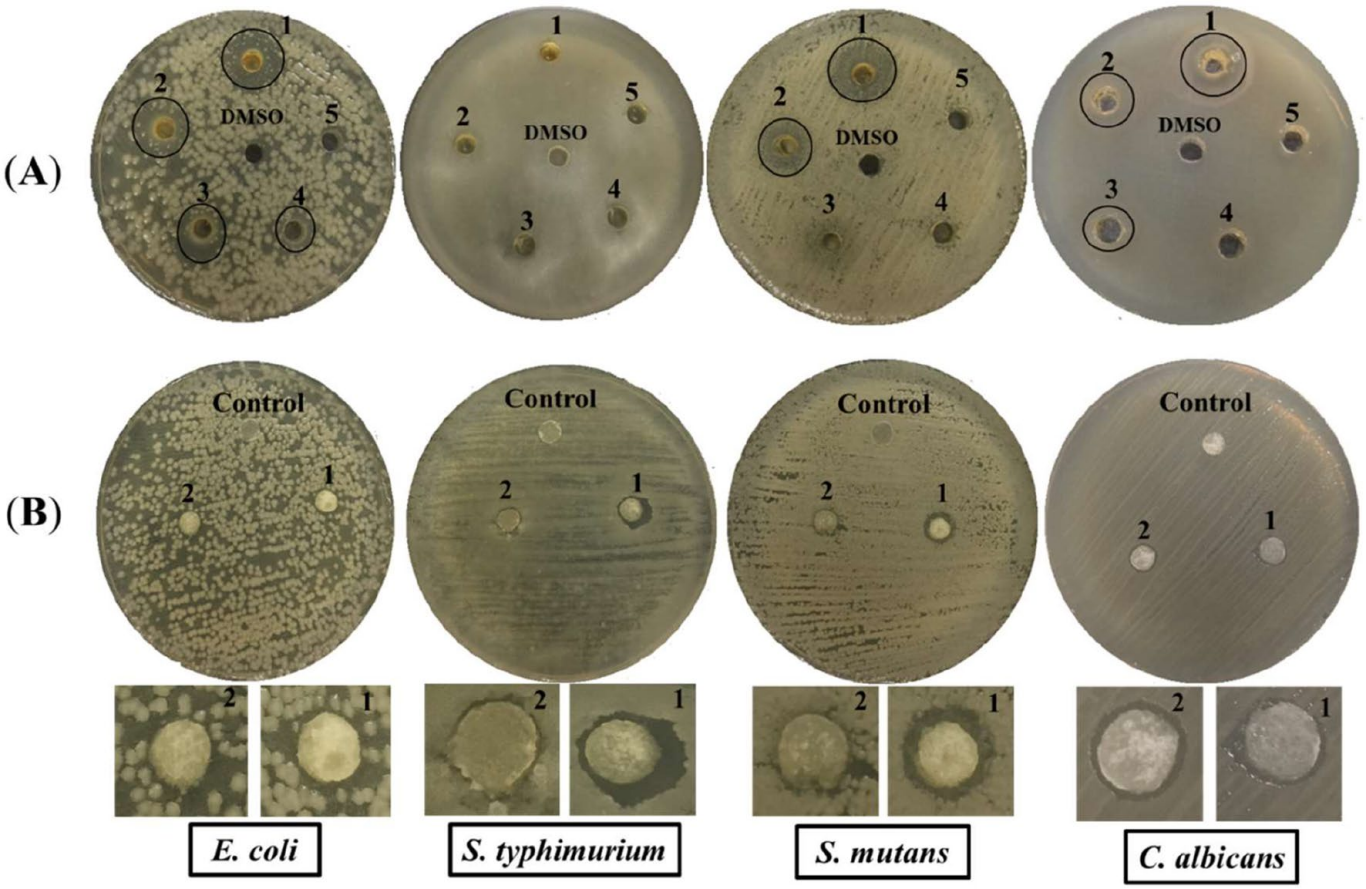
Antimicrobial activity expressed as halo-zones of the PE at different concentrations by agar well diffusion method (**A**) and CNF/PVA, CNF/PVA/PE1 and CNF/PVA/PE2 fiber reinforced hydrogels by disk diffusion method (**B**) against four pathogenic microbes.

Propolis is a resinous material extracted from the buds and flowers of plants and used as a protective agent against microbial contamination^42^. Polyphenols are the major chemical components of PE, and they are responsible for its antimicrobial activity^43^. The antimicrobial action of PE and CNF/PVA, CNF/PVA/PE1, and CNF/PVA/PE2 fiber reinforced hydrogels was explored on pathogens for humans using different approaches. The antimicrobial activity of PE is presented in Table 3 and Fig. 7. Although, PE exhibited antimicrobial potential against all of the studied microorganisms, it has no influence on *S. typhimurium* as reported by Atik et al.^44^. Control CNF/PVA hydrogel has no effect against the studied microorganisms. The results exhibit a significantly higher antimicrobial activity against Gram-positive (*S. mutans*), with no significant difference between Gram-negative (*E. coli*) and *C. albicans*. Moreover, the MIC was also determined through the investigation of antimicrobial activity at different concentrations. The MIC of PE for *E. coli* was 0.012 mg/mL, 0.05 mg/mL for *S. mutans*, and 0.025 mg/mL for *C. albicans*. On the other hand, the CNF/PVA fiber reinforced hydrogels with PE at different concentrations exhibit antimicrobial action against the strains used, with the highest antimicrobial activity being observed in *S. typhimurium*, followed by *S. mutans*, and other microbes showing no significant differences. The difference in antimicrobial behavior of the PE could be due to the different cell walls between the Gram-positive and negative bacteria^45^. The activity of PE as antimicrobial agents is related to the high percent of hesperetin (183.7 µg/mL), chlorogenic acid (96.9 µg/mL) and caffeic acid (90.2 µg/mL) as the main polyphenol compounds of PE. As illustrated in Table 1, hesperetin, chlorogenic acid, and caffeic acid were reported as three compounds from PE with high concentrations. Hesperetin was obtained from the enzymatic hydrolysis of hesperidin and evaluated for antimicrobial activity against Gram-positive and Gram-negative bacteria, the results revealed that hesperetin more effectively inhibited the model microbes than hesperidin and hesperidin glucoside ^46^. Yen et al.^47^. found that chlorogenic acid was one of the main components of the water extract of green coffee beans under high pressure and demonstrated antibacterial activity against both Gram-positive (*Staphylococcus aureus* and *Listeria innocua*) and Gram-negative (*Escherichia coli* and *Salmonella enterica*). Caffeic acid and its combination with antibiotic phytochemicals were evaluated against *Staphylococcus aureus,* and the results showed diverse effects against *Staphylococcus aureus,* with the MIC varying from 256 to 1024 µg/mL^48^. Most of the tested polyphenol compounds were not effective against the model microbes used when tested as a single compound, but when combined with other compounds, the activity of the antimicrobials increased. According to Ahmed et al.^49^ gallic acid at 200 and 400 µg/mL was effective against *S. aureus* and *S. pyogenes*, but not against Gram-negative bacteria. On the other hand, the antisolvent extracted from olive mill wastewater combined with gallic acid was tested using low minimal inhibitory concentrations, which revealed that 50/100–100/100 µg/mL caused complete growth inhibition of all the bacteria used, owing to the synergistic effects of phenolic compounds against the model bacteria used. Other study report that polyphenol compounds extracted from grape pomace combined with representatives of different classes of antibiotics such as β-lactam, quinolone, fluoroquinolone, tetracycline, and amphenicol act in synergy in all *S*. *aureus* and *E*. *coli* strains tested with fractional inhibitory concentration index (FICI) values varying from 0.031 to 0.155. The MIC was reduced 4 to 75 times due to the synergistic effects of phenolic compounds with different antibiotics^50^. Previous studies promoted the composite membrane by propolis to improve their biological assessments, for example polylactic acid/essential oil^51^, poly-ε-caprolactone^52^, bacterial cellulose/ZnO-NPs^53^, chitosan/Ag-NPs^54^, and polyurethane/ nanolignin^55^. We can summarize the main bioactive polyphenol compounds from PE as having antimicrobial activity in Table 4.

**Table 3:** The antimicrobial activity of the PE at five concentrations and CNF/PVA hydrogel reinforced with PE at different concentrations.

Organisms	Diameters of inhibition zone (mm)								
	PE (mg/mL)						Fibres reinforced hydrogels		
	DMSO	0.1	0.05	0.025	0.012	0.006	CNF/PVA	CNF/PVA/PE1	CNF/PVA/PE2
*E. coli*	0.0 ± 0.0	18 ± 0.01	15 ± 0.18	13 ± 1.05	10 ± 1.09	0.0 ± 0.0	0.0 ± 0.0	6 ± 0.1	5 ± 0.21
*S. typhimurium*	0.0 ± 0.0	0.0 ± 0.0	0.0 ± 0.0	0.0 ± 0.0	0.0 ± 0.0	0.0 ± 0.0	0.0 ± 0.0	11 ± 0.32	6 ± 0.34
*S. mutans*	0.0 ± 0.0	19 ± 1.02	16 ± 1.98	0.0 ± 0.0	0.0 ± 0.0	0.0 ± 0.0	0.0 ± 0.0	9 ± 0.41	5 ± 0.53
*C. albicans*	0.0 ± 0.0	16 ± 0.0	15 ± 0.0	11 ± 0.0	0.0 ± 0.0	0.0 ± 0.0	0.0 ± 0.0	7 ± 0.25	6 ± 0.43

All values were expressed as mean ± standard deviation.

**Table 4:** Antimicrobial activity of polyphenol compounds obtained from PE.

Polyphenol compound	Antimicrobial against	Reference
Gallic acid	*Escherichia coli*, *Bacillus cereus*, *Salmonella senftenberg*, *Listeria monocytogenes*, *Listeria innocua*, *Aspergillus, flavus*, *Aspergillus parasiticus*, *Fusarium oxysporum* and *Candida albicans*	^56^
Chlorogenic acid	*Streptococcus mutans, Streptococcus sanguinis, Streptococcus salivarius and Lactobacillus casei*	^57^
Methyl gallate	*Staphyloccocus aureus, Escherichia coli,* and *Bacillus cereus*	^58^
Caffeic acid	*Staphylococcus aureus*	^59^
Syringic acid	*Cronobacter sakazakii*	^60^
Pyro catechol	*Staphylococcus epidermidis*	^61^
Rutin	*Escherichia coli*, *Klebsiella pneumoniae* and, *Staphylococcus aureus*	^62^
Ellagic acid	*Staphylococcus aureus* and *Pseudomonas aeruginosa*	^63^
Coumaric acid	*Staphylococcus aureus*, *Bacillus subtilis*, *Escherichia coli*, *Candida albicans* and *Aspergillus niger*	^64^
Vanillin	*Escherichia coli*	^65^
Ferulic acid	*Listeria monocytogenes*	^66^
Naringenin	*Enterococcus faecalis*, *Staphylococcus aureus*, *Acinetobacter baumannii*, *Klebsiella pneumoniae* and *Helicobacter pylori*	^67^
Daidzein	*Acinetobacter baumannii*, *Escherichia coli*, *Klebsiella pneumoniae*, *Pseudomonas aeruginosa* and *Staphylococcus aureus*	^68^
Quercetin	*Staphylococcus aureus* and *Escherichia coli*	^69^
Cinnamic acid	*Aspergillus flavus*	^70^
Apigenin	*Staphylococcus aureus* and *Candida albicans*	^71^
Kaempferol	*Enterococcus faecalis*	^72^
Hesperetin	*Staphylococcus aureus*, *Bacillus cereus*,* Escherichia coli*, and *Pseudomonas aeruginosa*	^46^

### Cell viability

**Figure 8:**
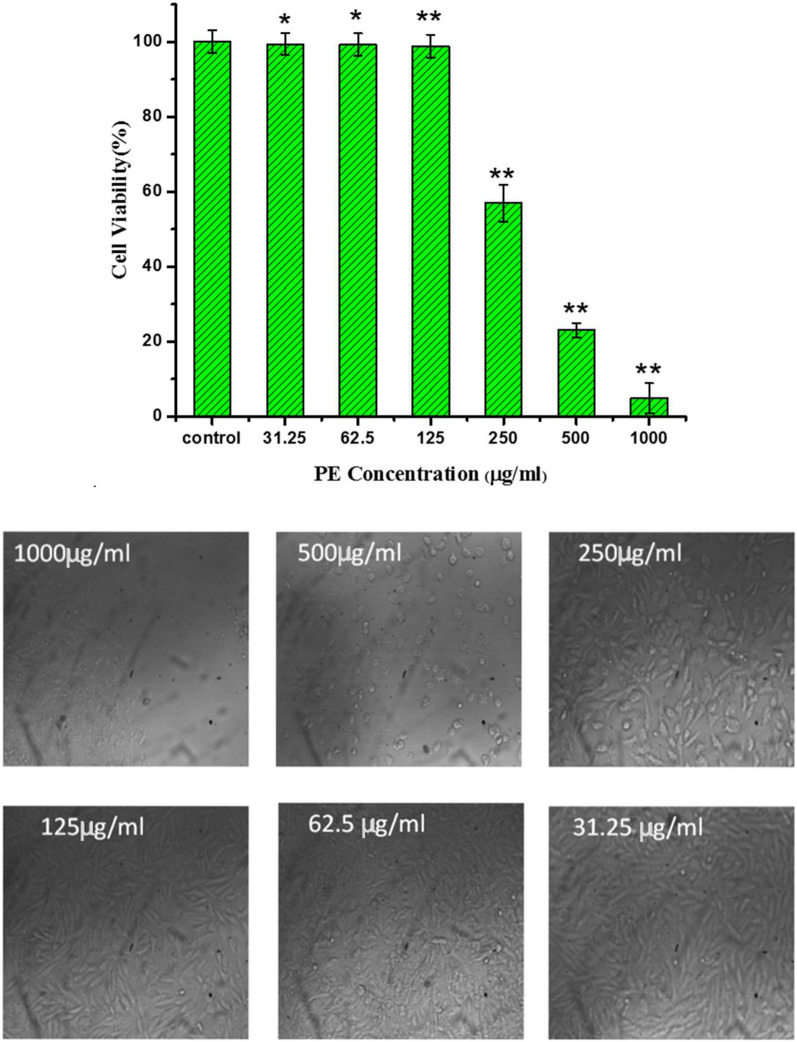
Viability of HFB4 cells treated with different concentrations (31.25–1000 μg/mL) of PE for 24 h. Data were expressed as the mean percentage of the formazan formation in untreated cells (control). Data were obtained from three independent experiments (six replicates for each experiment). (*) means p less than 0.05 and **p more than 0.05 compared to the control.

HFB4 cells were incubated with PE for 24 h at 37 °C in medium to develop a complete monolayer sheet. Different concentrations of PEs were applied up to concentrations of 1000 µg/mL. We observed that PE at concentrations greater than 125 µg/mL significantly reduced cell viability according to MTT (Fig. 8). Concentrations up to 125 µg/mL did not affect cell viability. Furthermore, light microscopy was used to confirm the cellular proliferation upon treatment of HFB4 cells with different doses of PEs. A decrease of cell number upon increasing PE concentration was clearly seen as compared to the control (Fig. 8). These results indicate that concentrations of PEs beyond the limit of 125 µg/mL may induce harmful effects in HFB4 cells. Previous studies demonstrated that supplementation with natural extracts may increase reactive species production and oxidative stress in vitro and in vivo tests, leading to oxidative damage and cell death^73,74^.

**Figure 9:**
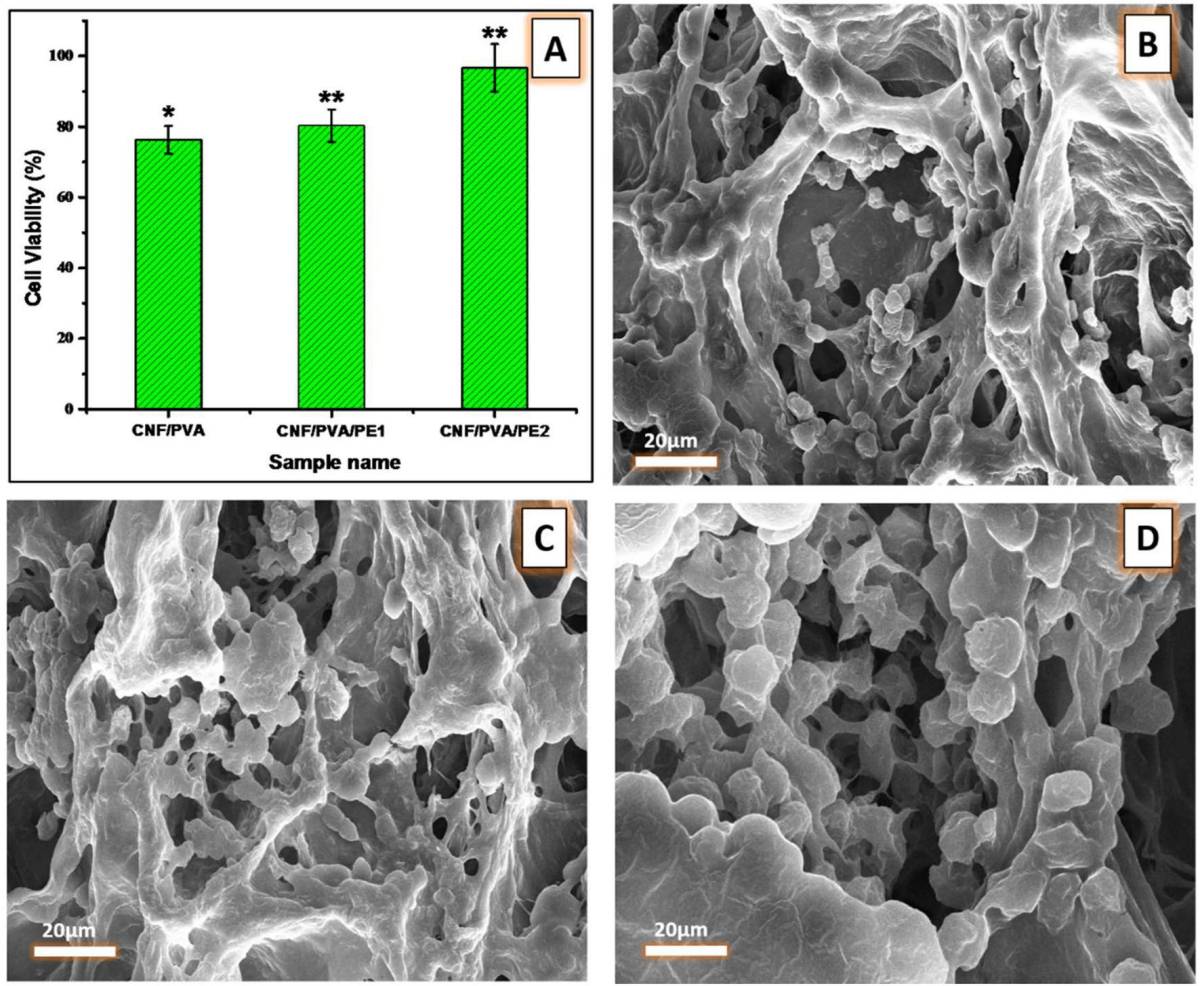
(**A**) Cell viability using MTT assay, while (**B**–**D**) SEM micrographs of HFB4 cells after 24 h seeded on CNF/PVA, CNF/PVA/PE1, and CNF/PVA/PE2 fiber-reinforced hydrogels, respectively. (*) means p less than 0.05 and **p more than 0.05 compared to the control.

Figure 9 shows the viability of HFB4 cells as determined by the MTT assay when treated with different samples of hydrogels. The viability of cells treated with samples containing PE (CNF/PVA/PE1 and CNF/PVA/PE2) exceeds than CNF/PVA sample without PE. This reflects that the presence of PE enhances the viability of HFB4 cells.

SEM images of the spreading and adhesion of HFB4 cells treated with CNF/PVA, CNF/PVA/PE1, and CNF/PVA/PE2 fiber reinforced hydrogels are shown in Fig. 9. As seen, all seeded cells have a rounded shape, which may be caused by the dehydration during the fixation process. The spreading of cells on samples loaded with propolis (CNF/PVA/PE1 and CNF/PVA/PE2) is higher compared to a control sample without PE (CNF/PVA). Also, the spreading and attachment of cells in the sample with a higher content of PE (CNF/PVA/PE2) is greater than in the sample with a lower content of PE (CNF/PVA/PE1). Therefore, the presence of propolis enhances cell growth and adhesion. Overall, the in vitro tests performed in this work illustrate the importance of the prepared propolis-reinforced hydrogel as a cytocompatible material promoting cell adhesion and spreading in a 3D environment.

### In vitro anti-inflammatory tests (hemolysis inhibition)

The stabilization of erythrocyte membranes is an established assay to investigate the anti-inflammatory effect of implanted biomaterials. Inflammation occurs due to release of different enzymes from decomposed lysosomal vesicles^75^. Therefore, stabilization of erythrocyte membrane prevents leakage of these enzymes, as well as incidence of inflammation^75^. Hemolysis assay is very critical assay especially for materials that will be in contact with the blood. Table 5 explains the results of hemolysis inhibition for PE, samples of CNF/PVA hydrogel and those loaded with propolis (CNF/PVA/PE1 and CNF/PVA/PE2). The indomethacin drug was used as the slandered (positive control) and it has the highest protection (100 ± 2.362%) at a concentration of 200 µg/mL against hypotonicity induced lysis of RBCs. As shown in Table 2, all samples protect human erythrocytes in a concentration dependent manner. The protective effect of CNF/PVA/PE2 sample and PE is almost the same at different concentrations. The lowest protective effect is recorded for the CNF/PVA sample (without PE). From these findings, it’s clear that, the PE loaded hydrogels are promising as anti-inflammatory agents.

**Table 5:** Anti-inflammatory assay of PE and CNF/PVA composite reinforced with PE at different concentrations.

Hemolysis inhibition (%) at different concentrations						
Sample	1000 (µg/mL)	800 (µg/mL)	600 (µg/mL)	400 (µg/mL)	200 (µg/mL)	100 (µg/mL)
PE	88.8 ± 1.312	83.4 ± 1.314	80.5 ± 2.213	76.2 ± 1.523	74.9 ± 2.319	72.6 ± 1.942
CNF/PVA	79.4 ± 2.002	76.1 ± 2.504	72.3 ± 2.003	69.8 ± 1.803	66.9 ± 2.009	64.3 ± 3.104
CNF/PVA/PE1	82.1 ± 2.002	80.7 ± 1.004	78.4 ± 1.603	75.3 ± 3.003	72.2 ± 3.309	68.4 ± 2.014
CNF/PVA/PE2	88.3 ± 2.472	85.2 ± 2.934	80.8 ± 2.603	78.5 ± 3.053	74.1 ± 1.029	70.2 ± 3.004
Positive control	100 ± 2.362					

All values were expressed as mean ± standard deviation.

## Conclusion

Propolis is a natural byproduct with high value for diverse biological applications. The HPLC analyses of PE revealed about 18 polyphenol compounds, with the highest concentration being hesperetin, followed by chlorogenic acid and caffeic acid. CNF was prepared by oxidation-defibrillation of cellulose obtained from bleached sugarcane bagasse, and used to prepare sustainable and porous CNF/PVA loaded with various PE concentrations. The prepared scaffolds exhibited a high porous structure with pore sizes up to 100 µm. The obtained PE-loaded CNF/PVA hydrogels have a highly antimicrobial activity against *E. coli*, *S. mutans* bacteria and then *C. albicans*. The cellular responses also showed that the cells on the PE-functionalized CNF/PVA hydrogels revealed an optimal microenvironment for the attachment and proliferation of HFB4 cells. Remarkably, our results suggested that PE-functionalization of CNF/PVA not only enhanced the antimicrobial features of PE-loaded CNF/PVA hydrogels, but also resulted in significant anti-inflammation potential. Thus, in vivo experiments are highly recommended prior to clinical application to underline the potency of PE-loaded CNF/PVA hydrogels in wound healing.

## Materials and methods

### Materials

Bleached bagasse pulp was obtained from Qena Company of Paper Industry, Egypt. TEMPO was purchased from Merck. Sodium bromide was purchased from Sigma-Aldrich. The raw propolis material used in this work was collected from a local beekeeper (Mansoura, Dakahlia, Egypt) in July 2022. Polyvinyl alcohol—PVA (MW85,000–124,000 g/mol, 99.1% hydrolized) was purchased from Sigma-Aldrich. Sodium hydroxide, distilled water, and ethanol were also employed in this research.

### Preparation of PE

PE was collected according to Bozkuş et al.^31^ with little modifications. In brief, about 10 g of raw material was dissolved in 100 mL of 70% ethanol and then placed in an incubator at 37 °C for 14 days in a dark place. After filtration using Whatman filter paper (No. 1), the mixture was centrifuged at 5000 rpm for 15 min and then oven dried at 40 °C for 5 days until constant weight. The PE obtained was stored in the refrigerator until used.

### Evaluation of polyphenol compounds

The polyphenol (phenolic and flavonoids) compounds of PE were analyzed by HPLC (Agilent 1260 series, USA), according to Kong et al., with slight modifications^76^. Chromatographic conditions: the chromatographic separation was carried out on an Eclips C18 column (4.6 × 250 mm, 5.0 µm). The mobile phase comprised (A) water and (B) 0.05% trifluoroacetic acid in acetonitrile at a flow rate of 1 mL/min. The solvent gradient was: 0 min 82% A, 0–5 min 80% A, 5–8 min 60% A, 8–12 min 60% A, 12–15 min 82% A, 15–16 min 82% A, and 16–20 min 82% A. The multi-wavelength detector was monitored at 280 nm. The injecting volume was 5 µl for each of the sample solutions. The column temperature was maintained at 40 °C.

### Preparation of CNF

Bleached bagasse was used for the preparation of TEMPO-oxidized CNF by using the method described by Salama et al.^35^. In brief, 20 g of bleached bagasse pulp were dispersed in distilled water with TEMPO (0.8 g) and sodium bromide (8 g). Following this, 300 mL of sodium hypochlorite solution (15%) was continuously added and the pH was adjusted to 10 using NaOH (3 mol/L) solution. At the end of the reaction, the pH was reduced to 7.0 and the product was centrifuged several times at 10,000 rpm. Dialysis using deionized water was performed to purify the product.

### Preparation of CNF/PVA hydrogel reinforced by PE

The CNF/PVA porous hydrogel was prepared using the freeze-thawing and freeze drying methods according to Müller et al.^77^. The polymeric weight ratio was adjusted to 50:50 (CNF:PVA). In brief, 30 mL of PVA solution with a concentration of 10 wt% was prepared in distilled water using magnetic stirring at 95 °C. After 4 h, 100 mL of CNF solution (3 wt%) was added to PVA solution and underwent mechanical agitation for 10 min at 5000 rpm. Then, the CNF/PVA mixture was poured into a 10 cm round mould and freezen at − 40 °C for 3 h, followed by thawing at room temperature for 3 h to allow PVA crosslinking. The obtained hydrogels were subjected to four freeze-thawing cycles and subsequently freeze-dried at − 80 °C. To produce PE loaded CNF/PVA hydrogels, different proportions of the PE (0.1 and 0.2 g) were dissolved in 2 mL of ethanol and then added to 22 mL of the CNF/PVA suspension solution mixture. The mixtures were kept on a magnetic stirrer at 40 °C for 1 h to assure complete dissolution of PE. The solutions were homogenized using mechanical agitation for 10 min at 5000 rpm before being poured into round mold and subjected to freeze-thawing cycle and finally lyophilized at − 80 °C, as mentioned above. The prepared samples were named CNF/PVA for control (without PE), CNF/PVA/PE1 for sample with 0.1 g PE and CNF/PVA/PE2 for sample with 0.2 g PE.

### Characterizations of composite hydrogel

#### Structure and morphological analysis

The morphology of the prepared CNF/PVA hydrogel reinforced with different concentrations of PE was examined using field emission scanning electron microscopy (FE-SEM) (JSM 6360LV, JEOL/Noran). The estimation of pore size was determined from different SEM images using the angle tool of ImageJ with Java 1.8.0 software (National Institute of Health (NIH), USA)^78^. The FT-IR (model FT/IR-6100 type A) was used to investigate the chemical functional groups of the samples. The spectra were recorded at wave numbers in the range of 400–4000 cm^−1^. The XRD pattern was recorded with an empyrean powder diffractometer (Cu Kα, 0.154 nm) between 5 and 70° 2θ with a step size of 0.01°/s to examine the phase formed in the prepared CNF. Ultra High-Resolution Transmission Electron Microscope (JEOL-2010) was applied to examine the internal structure of CNF.

#### Water uptake ratios

The evaluation of prepared hydrogel for water uptake was carried out according to Eskandarinia et al.^79^ with slight changes. Prepared fiber reinforced hydrogels (CNF/PVA, CNF/PVA/PE1, and CNF/PVA/PE2) were cut into 1 cm × 1 cm, accurately weighed (W_i_), and immersed in 10 mL of distilled water in sealed vials at 37 °C. At scheduled time intervals (1, 2, 3, 4, 5, 6, 8, and 12 h), samples were withdrawn from the vials, blotted with a tissue paper to remove the surface water and weighed (W_w_). The water uptake (swelling) ratio of the samples is specified according to the following equation:

$$\mathrm{water \; uptake \; ratio}=\frac{{\mathrm{W}}_{\mathrm{w}}-{\mathrm{W}}_{\mathrm{i}}}{{\mathrm{W}}_{\mathrm{i}}} \times 100$$

### Biological applications of PE and composite hydrogel

#### Antimicrobial activity

##### Microorganism and growth conditions

The antimicrobial activity of the PE, CNF/PVA, CNF/PVA/PE1 and CNF/PVA/PE2 fiber reinforced hydrogels was assessed against four selected pathogens, including Gram-negative bacteria [*Escherichia coli* ATCC 25922 (*E. coli*) and *Salmonella* *typhimurium *ATCC 14028 (*S. typhimurium*)]*,* Gram-positive bacteria [*Streptococcus mutans* ATCC 25175 (*S. mutans*)], and yeast [*Candida albicans* ATCC 10231 (*C. albicans*)]. All microbes were provided by American Type Culture Collection (ATCC). The microbial strains were cultivated in a test tube containing 5 mL of Mueller Hinton broth composed of (%): 0.2 beef extract, 1.75 acid hydrolysate of casein, and 0.15 starch, under staring cultivation (200 rpm) for 1 day at 37 °C. The antimicrobial activity of the propolis and different hydrogel samples were carried out through the agar well and disk diffusion approach, respectively.

##### Agar well diffusion technique

The antimicrobial action of the PE was evaluated qualitatively by the agar well diffusion technique according to Roy and Rhim^80^, with little modification. Shortly, the microbial suspension (10^8^ CFU/mL) with approximately equal concentration or density with 0.5 McFarland standards was spread on the Petri dish surface containing Mueller Hinton agar medium (2%). The minimum inhibitory concentration (MIC) is the minimum concentration of the PE that shows a low inhibition zone against the microbial target. For MIC determination, the propolis at different concentrations of 0.1, 0.05, 0.025, 0.012, and 0.006 mg/mL (labelled as 1, 2, 3, 4 and 5, respectively) was dug in the media wells (4 mm in diameter) by a sterile glassy borer and loaded with 65 μL of tested propolis. The negative control was performed by loading the center wells with DMSO.

##### Disk diffusion technique

The antimicrobial activity of the prepared CNF/PVA, CNF/PVA/PE1, and CNF/PVA/PE2 fiber reinforced hydrogels was carried out through the disk diffusion approach according to Yahia et al.^81^, with minor modifications. In brief, 100 µL of serially diluted pathogens were separately distributed on the Petri dishes of Mueller Hinton agar medium along with disks (5 mm in diameter) of the composite membranes loaded with different concentrations of propolis. The fiber reinforced hydrogel without propolis was used as a control.

##### Incubation conditions and assay

All the plates were left at 4 °C for 120 min. to complete the diffusion of the tested sample as well as inhibit the model microbes, subsequently incubated at 37 °C for 1 day, where the antibacterial activity was assessed by measuring the developed inhibition-zone diameter (including the well or membrane disk) after the incubation period. To ensure the aseptic conditions, all membranes were sterilized for 30 min under UV light prior to application. All experiments were conducted in triplicate, and the mean results were represented.

##### Cytotoxicity assay

Cytotoxicity assay of CNF/PVA, CNF/PVA/PE1, and CNF/PVA/PE2 fiber reinforced hydrogels and also PE against HFB4 cells was performed using the MTT (3-4,5-dimethyl-2-thiazolyl)-2,5-diphenyl-2H-tetrazolium bromide) assay^82,83^. The prepared hydrogels were rinsed with media three times and then plated in the bottom of a 24 well tissue culture plate. The 24 well plate was inoculated with 1 × 10^5^ cells/mL (1000 µL/well) and incubated at 37 °C for 24 h to develop a complete monolayer sheet. Cells were observed for sheet formation and cytotoxicity effects. The HFB4 cells were checked for any physical signs of toxicity, e.g., complete or partial loss of the monolayer, rounding, shrinkage, or cell granulation. MTT solution was prepared (5 mg/mL in PBS) (BioBasic Canada Inc). 100 µL MTT solution was appended to each well. Put on a shaking table, 150 rpm for 5 min, to thoroughly blend the MTT into the media. Incubate (37 °C, 5% CO_2_) for 4 h to allow the MTT to be metabolized. After that, 200 μL of DMSO was mixed with the culture cells to solubilize the formazan crystals, which were obtained via the reduction of MTT in living cells. Subsequently, the mean absorbance of each well at 570 nm was calculated using a spectrophotometer. Three parallel experiments were performed. The absorbance should be directly correlated with cell quantity^82,83^. For PE, the 24 well tissue culture plate was inoculated with 1 × 10^5^ cells/mL (100 µL/well) and incubated at 37 °C for 24 h to develop a complete monolayer sheet. Growth medium was decanted from 24 well microtiter plates after a confluent sheet of cells was formed. Then the cell monolayer was washed twice with wash media. A twofold dilution of the tested sample was made in RPMI medium with 2% serum (maintenance medium). 0.1 mL of each dilution was tested in different wells, leaving 3 wells as controls, receiving only maintenance medium. Then the same steps were performed as mentioned above.

##### Cell adhesion

HFB4 cells were grown in 24-well plates containing CNF/PVA, CNF/PVA/PE1, and CNF/PVA/PE2 fiber reinforced hydrogels^84^. After 24 h incubation, the cells were fixed with 5% (V/V) glutaraldehyde and then dehydrated gradually with serial solutions of ethanol (70–100% for 20 min each). After that, the samples sputtered with gold for SEM observations.

### Anti-inflammatory assay

The preparation of erythrocyte suspension was done according to Anosike et al.^75^. In brief, about 3 mL of fresh whole blood gathered from healthy volunteers was placed in heparinized tubes and then centrifuged at 3000 rpm for 10 min. A volume of normal saline equal to that of the supernatant was utilized to dissolve the red blood pellets. The volume of the obtained dissolved red blood pellets was measured and returned as a 40% v/v suspension with an isotonic buffer solution (10 mM sodium phosphate buffer, pH 7.4). The buffer solution was formed from 0.2 g of NaH_2_PO_4_, 1.15 g of Na_2_HPO_4_ and 9 g of NaCl in 1 L of distilled water. The returned red blood cells (resuspended supernatant) were used as such.

#### Hypotonicity induced hemolysis

Equal weights of CNF/PVA, CNF/PVA/PE1, and CNF/PVA/PE2 fiber reinforced hydrogels were ground carefully and suspended in distilled water (hypotonic solution). 5 mL of the hypotonic solution of graded doses of the samples (100, 200, 400, 600, 800, and 1000 µg/mL) were placed into duplicate pairs (per dose) of the centrifuge tubes. 5 mL of isotonic solution of graded doses of the samples (100–1000 µg/mL) were also placed into duplicate pairs (per dose) of the centrifuge tubes. The same previous steps were occurred for PE resin, but without grinding. A tube containing 5 mL of the vehicle (distilled water) and another tube containing 5 mL of 200 µg/mL of indomethacin were used as the control tubes, respectively. A suspension (0.1 mL) of erythrocytes was added to each of the tubes and mixed gently. The mixtures were incubated for 1 h at room temperature (37 °C), and afterwards, they were centrifuged for 3 min at 1300 rpm. The absorbance (Ab) of hemoglobin within the supernatant was estimated at 540 nm using a Spectronic (Milton Roy) spectrophotometer. To calculate the hemolysis percentage, the hemolysis formed in the presence of distilled water was assumed to be 100%. The percent inhibition of hemolysis by the extract was calculated thus:

$$\% {\text{Inhibition of haemolysis}} = {1} - \left( {\left( {{\text{Ab}}_{{2}} - {\text{Ab}}_{{1}} } \right)/ \, \left( {{\text{Ab}}_{{3}} - {\text{Ab}}_{{1}} } \right)} \right)*{1}00$$

where Ab_1_ = absorbance of test sample in isotonic solution, Ab_2_ = absorbance of test sample in hypotonic solution, Ab_3_ = absorbance of control sample in hypotonic solution^75,85^.

### Statistical analysis

All experiments were done in triplicate, and the results were presented as mean ± standard deviation. Statistical analyses were performed with the one-way ANOVA test, by using Sigma Stat 3.5 software (Dundas Software Ltd, Toronto; Canada). P values ≤ 0.05 were considered statistically significant.

## Data Availability

The datasets used and/or analyzed during the current study are available from the corresponding author on reasonable request.
